# Dr. Cuming, on the Use of Nitre in Sphacelus

**Published:** 1806-06

**Authors:** Ralph Cuming


### *To the Editors of the Medical and Physical Journal.*

Gentlemen,

I Have to acknowledge my obligation to Mr. Simpson of Skipton, and also to Mr. Shearly at the Royal Naval Hospital at Deal, for the testimony which they have lately bore in confirmation of the truth of my assertions, some time since made in the Medical and Physical Journal, respecting the superior excellence and power, which nitre can boast of over every remedy that has hitherto been used in the cure of sphacelus. Mr. Simpson observes, "suffice to it say, the whole class of stimulants and antiseptics, were tried internally and externally, notwithstanding the putrid diathesis increased daily, until arrested by this sovereign remedy," he concludes by joining me in recommending this invaluable medicine to the public.

With regard to Mr. Shearly's observations, I must beg leave to make a few comments, and to apprise him of an error he has committed in stating, that I recommended nitre in gun-shot wounds; this is an honour I must disclaim; it is true, that the first time I thought of it, was in a case of mortification originating from a gun-shot wound, since which, the same happy result has arisen from several cases of sphacelus that have come under my care and cognizance; more of which would, in all probability, have been published, had not the fear of criticism, and the intemperate remarks which are so frequently made, together with that polite language and supreme urbanity which characterize these snarling productions, operated as preventatives.

Though

Though, in all gun-shot wounds sloughing takes place, and this slough may be considered as a partial mortification, yet the common routine of practice will, aided by the efforts of Nature, in general cast it off. The propriety of the application of nitre in the early stages of such wounds is doubtful, when almost every danger to be apprehended arises from inflammation: but, on the other hand, when the efforts of Nature begin to flag, inflammation vanishes, and much fœtor, with want of tone in the part, are prevalent, its utility is unquestionably great. Mr. Shearly remarks, "that previous to seeing Dr. Cuming's remark on the great utility of nitre in gun-shot wounds, I had witnessed its great efficacy in cleansing ulcers degenerating from a healthy into a sloughy state." Whatever Mr. S. may have witnessed of its effects as a detergent, I believe he is a stranger to its modus operandi in sphacelus, and that I am the first person who ever used it and recommended it in such cases. Mr. S. says, "The greatest power which nitre seems to possess, is its accelerating the separation of the sloughs, and by assisting Nature in her operations. But another property which Dr. Cuming has ascbribed to it, I have never detected, namely, its correcting the fœtor which emanates in a great degree from this species of wounds."

That nitre assists Nature in her operations is evident by its stimulant power; but what astonishes me most, is, that Mr. Shearly should appear to imagine that it does not possess antiseptic properties, as he does not subscribe to its efficacy in correcting fœtor; for my own part, I conceive it to be one of the first antiseptics in all nature: but how should he have experienced that effect from it, which is its peculiar characteristic, and which I have dwelt upon so much in my communications respecting it, when he has only used it in the form of a weak lotion? the power of which must have been transient, for it does not appear that lint was immersed in it and left applied to the wound ; for, in the case he records, the wound was washed with the solution night and morning, and covered by charpeé. Undoubtedly, different circumstances point out the propriety of a variety of modifications in the application of this remedy; yet in all cases of sphacelus it will be absolutely requisite to apply it in the manner I have recommended, viz. by sprinkling the powder over the part, and by completely covering it; its thickness being in a ratio proportionate to the extent of the disease, retaining it with suitable dressings. Whoever uses nitre in the above way, will find my assertions respecting its effects to rest on the basis of truth, supported by experience; it not only subdues fœtor, but, as I have elsewhere observed, preserves the system from the absorption of putrid virus, by rendering the sphacelated parts innocuous. I have, in a medical work with which I have lately been engaged, treated on the subject of sphacelus, at some length, from which the following query is a quotation

May not nitre, conjoined with aromatics, hereafter prove beneficial in putrid fevers? Inferences deduced from the above narrations, are greatly in favour of the pleasing expectation.

I am, &c.

*April* 25, 1806. RALPH CUMING, M. D

